# Necrotizing Enterocolitis and Intestinal Microbiota: The Timing of Disease and Combined Effects of Multiple Species

**DOI:** 10.3389/fped.2021.657349

**Published:** 2021-05-03

**Authors:** Xin Fu, Siwen Li, Yanfang Jiang, Xintong Hu, Hui Wu

**Affiliations:** ^1^Department of Neonatology, The First Hospital of Jilin University, Changchun, China; ^2^Gene Diagnosis Center, The First Hospital of Jilin University, Changchun, China

**Keywords:** necrotizing enterocolitis, intestinal microbiota, 16S rRNA, premature infants, newborn, risk factors

## Abstract

**Background:** The purpose of this study was to investigate the relationship between intestinal microbiota and necrotizing enterocolitis (NEC).

**Methods:** 16S rRNA gene sequencing was used to compare the microbial composition of feces. The first sample was collected within 48 h after birth, then once per week until the NEC diagnosis, and finally 1–2 weeks after treatment or 28 days after birth.

**Results:** The alpha diversity of the microbiota in the NEC group was higher than that in the control group. Beta diversity analysis showed that the control group had a higher similarity at the onset of NEC, while the NEC group was distributed in subgroups. Linear discriminant analysis effect size and taxonomic composition analyses indicated that the abundance of Bacteroides and Actinobacteria in NEC infants at birth was much higher than that in the control group, and this trend continued until NEC occurred. At this time, Rhizobiales, Dysgonomonas, Ochrobactrum, Ralstonia, Pelomonas, Acinetobacter, etc., were also more abundant in NEC infants. The upregulated different metabolic pathways in the NEC group were mainly concentrated on degradation/utilization/assimilation, biosynthesis, and generation of precursor metabolites and energy.

**Conclusions**:

1. The microbial community differs according to the time of NEC diagnosis (bounded by 20 days).

2. No single microorganism is related to NEC, and the combined effect of multiple species is of great significance in the occurrence of NEC. Premature infants are easily affected by bacteria living in the environment, and compared with ordinary premature infants, NEC infants have a higher abundance of waterborne bacteria. Therefore, attention should be paid to the contamination of water sources and various ventilator pipelines for premature infants hospitalized in the neonatal intensive care unit.

3. An in-depth study of the mode of microbial colonization in premature infants combined with the different functions of various metabolic pathways involved in different microorganisms may be able to identify the cause of NEC.

## Introduction

Necrotizing enterocolitis (NEC) is one of the most common gastrointestinal emergencies in newborns and mostly occurs in premature infants born before 34 weeks of gestation. Abdominal distension, vomiting, diarrhea, hematochezia, shock, and multiple organ system failure in severe cases are the main clinical manifestations. In the United States, the incidence of proven or severe NEC (Bell stage II and III) is estimated to be ~1–3 per 1,000 live births (1, 2).

As a typical intestinal infectious disease, in 1975, Sántulli et al. hypothesized that microbial imbalance in the digestive tract is involved in the pathogenesis of NEC, and this condition is dominated by bacterial disorders (3). This imbalance is manifested by the lack of colonization by certain normal bacteria in the intestine or the occurrence of abnormal colonization, which is regarded as a key risk factor in NEC (4). With the development of high-throughput sequencing, researchers can identify more abundant bacterial communities and have a deeper understanding of the relationship between NEC and gut microbes.

Current research not only focuses on the impact of individual microbes on the disease but also regards gut microbes as a whole. In addition to discovering the relationship between specific taxa such as Firmicutes, Proteobacteria, Klebsiella, and Actinomycetes and NEC, microbial diversity has been found to be associated with diseases. Because of the low diversity of intestinal microbes in early life, large individual differences, and diverse sequencing platforms, no specific species or uniform microbial characteristics have been confirmed as having a causal relationship with NEC. In the meantime, there are currently few studies on the flora characteristics of infants with NEC in Asia, especially in China. This study focused on infants from Jilin Province, China. In addition to analyzing the characteristics of intestinal flora during the occurrence of NEC, the meconium of infants was analyzed within 48 h after birth to explore the overall characteristics of the fecal flora of infants with NEC and its different flora. Understanding the intestinal microbiota can help us take measures to change the outcome of infants before the disease occurs.

## Materials and Methods

This study was conducted from February 2018 to April 2019 and approved by the Ethics Committee of the First Hospital of Jilin University (Ethics No. 2018-421).

### Inclusion Criteria

1. Gestational age ≤ 33 weeks
2. Premature infants with incomplete meconium excretion (within 48 h after birth)

### Exclusion Criteria

1. Congenital intestinal malformations and other serious malformations, such as fetal gastroschisis and intestinal atresia
2. Spontaneous intestinal perforation
3. Probiotic usage before the first stool specimen collection
4. Incomplete clinical data or failure to follow the procedures to store samples

### Sample Collection Process

All fecal samples were collected within 48 h of birth of premature infants who met the inclusion criteria and then once a week. When an infant presented with NEC symptoms and was diagnosed with NEC (Bell stage II and III), they were classified into the NEC group, and stool samples were taken within 48 h. Next, stool samples were collected 1–2 weeks after treatment from premature infants with confirmed NEC. Premature infants without NEC would continue to provide weekly stool samples until they were discharged from the hospital. All infants left the study because of death and treatment abandonment. The samples were stored at −20°C within 24 h after collection and then transferred to −80°C for low-temperature storage until being sent to Shanghai Parsons Biotechnology Co., Ltd., for 16sRNA analysis.

### Sample Size Selection and Control Group Matching

To exclude confounding factors, infants without NEC who matched the infants with NEC in gestational age, delivery mode, feeding patterns, antibiotic application time, and other diseases were enrolled as controls. In the early stage of the study, 10 samples (10 in the control group and 10 in the NEC group) were tested for intestinal flora analysis, and the mean value and standard deviation of the alpha diversity index were obtained. Then, the values were imported into MedCalc software for sample size calculation. The results showed that, with α = 0.05, β = 0.01, 80% power, and 95% confidence level, the required sample size was 13 in the NEC group and 13 in the control group. We appropriately raised the sample number to 15 cases.

### PCR Amplification and Illumina NovaSeq Sequencing

Total bacterial genomic DNA was extracted from fecal samples, and PCR amplification was performed on the V3–V4 region of the bacterial 16S rRNA gene. The forward primer was 338F (5′-ACTCCTACGGGAGGCAGCA-3′), and the reverse primer was 806R (5′-GGACTACHVGGGTWTCTAAT-3′). The Illumina NovaSeqPE250 DNA library was selected as the sequencing platform to perform paired-end sequencing of community DNA fragments, and the original sequencing data were saved in FASTQ format (raw data about the study have been uploaded to NCBI, PRJNA694799).

### Bioinformatic Processing

#### Sequence Data Analysis

Sequence data analysis was performed mainly with QIIME2 and R software (v3.2.0). The DADA2 algorithm was used to carry out depriming, quality filtering, denoising, splicing, and dechimerism; the algorithm no longer uses similarity clustering and only performs dereplication or an equivalent of 100% similarity clustering. After obtaining the amplicon sequence variant abundance matrix based on the Greengenes database, the bacterial flora between groups was analyzed.

#### Overall Analysis of the Fecal Community

Alpha diversity analysis was used to show the richness and diversity of the community, and a rarefaction curve was used to judge whether the current sequencing depth of each sample was sufficient to reflect the microbial diversity and community abundance contained in the community sample. The R script was used to visualize the alpha diversity index values as box plots. Beta diversity analysis was performed mainly by using a two-dimensional sequence diagram of the non-metric multidimensional scaling (NMDS) analysis results to show the differences in microbial community composition of each sample and combined with ANOSIM to reflect the community differences between groups and within each group. At the same time, taxonomic composition analysis was carried out to analyze the trend of species change within each group.

#### Different Flora and Metabolic Analysis

Linear discriminant analysis effect size (LEfSe) analysis was used to obtain the different flora between the groups, and taxonomic composition analysis was combined to identify the different flora with higher relative abundance. Using the PICRUSt2 method, the MetaCyc database (https://metacyc.org/) was compared to obtain different metabolic pathways between groups to predict the function of the microbial community.

#### Statistical Method

SPSS 22 software was used to analyze the clinical data of all participants. The measurement data conforming to normal distribution were expressed as mean ± standard deviation (*X* ± SD), and the comparison between groups was performed with *t*-test; a non-parametric test was used for abnormally distributed data. *P* < 0.05 indicated that the difference was statistically significant.

## Results

**Figure 1:**
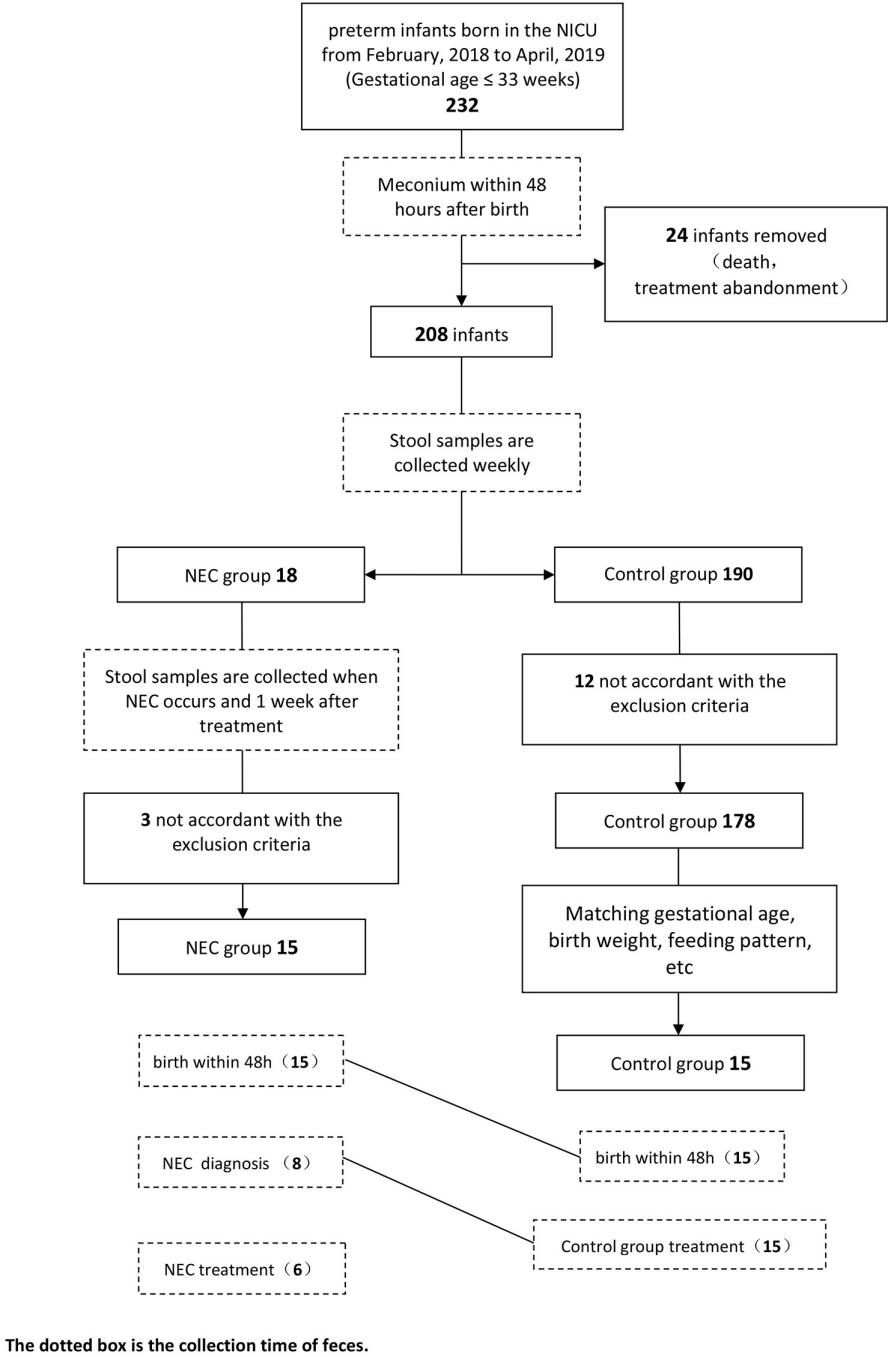
Flow diagram.

In this study, a total of 15 infants with NEC and 15 infants in the control group participated in the analysis. The specific screening process is shown in the flow diagram (Figure 1). Eight samples of infants were taken when NEC occurred. According to statistics, these eight samples were collected, on average, ~1.25 days after the onset of NEC symptoms, and the NEC diagnosis time was 10–27 days after birth. After NEC treatment, the samples of six infants were collected. The control group stool samples were collected within 48 h of birth and the time close to the diagnosis in the NEC group. All infants in the NEC group were treated with antibiotics according to the severity of the disease, and three of them underwent enterostomy. All infants were breastfeeding priority, and some of the infants were given formula feeding due to insufficient breast milk. Feeding intolerance often occurs in premature infants, so some infants use probiotics. *Clostridium butyricum* and Bifidobacterium are commonly used in our center. To reduce the differences between groups, we also matched the types of probiotics used. The basic characteristics of the two groups are shown in Table 1.

**Table 1:** Infant characteristics and clinical information.

	**Necrotizing enterocolitis (NEC) group (*n* = 15)** **Mean ± SD or *n* (%)**	**Control group (*n* = 15)** **Mean ± SD or *n* (%)**	***P*-value**
Gestational age (weeks)	30.2 ± 1.2	30.1 ± 1.9	0.92
Birth weight (kg)	1.46 ± 0.32	1.36 ± 0.37	0.41
Sex (male)	9 (60)	9 (60)	1.0
Delivery mode (vaginal)	7 (46.7)	8 (53.3)	1.0
**Feeding pattern**			
First stool (ambrosia/formula)	7/8	7/8	
Second stool (exclusive/mixed breastfeeding)	1/7	1/14	
Apgar score-−5 min	6.4 ± 1.7	6.3 ± 1.3	0.68
Probiotics	11 (73.3)	9 (60)	0.70
*Clostridium butyricum*	8	5	
Bifidobacterium	1	0	
Combined probiotics	2	4	
Use of antibiotics (days)	14.4 ± 6.8	9.7 ± 6.2	0.12
	Sulbenicillin, ceftriaxone, cefepime, meropenem, piperacillin-tazobactam	Sulbenicillin, cefoperazone, cefepime, vancomycin, piperacillin-tazobactam	
Premature rupture of membranes	2 (13.3)	5 (33.3)	0.39
Received surfactant	10 (66.7)	10 (66.7)	1.0
Received antenatal steroids	11 (73.3)	10 (66.7)	1.0
Received antenatal antibiotics	1 (6.7)	2 (13.3)	1.0
**Maternal factors**			
Gestational hypertension	5 (33.3)	4 (33.3)	1.0
Gestational diabetes	2 (13.3)	2 (13.3)	1.0

*Mean ± SD, mean ± standard deviation*.

*The duration of antibiotic use in the NEC group was before NEC diagnosis*.

### Overall Characteristics of the Fecal Flora

**Figure 2:**
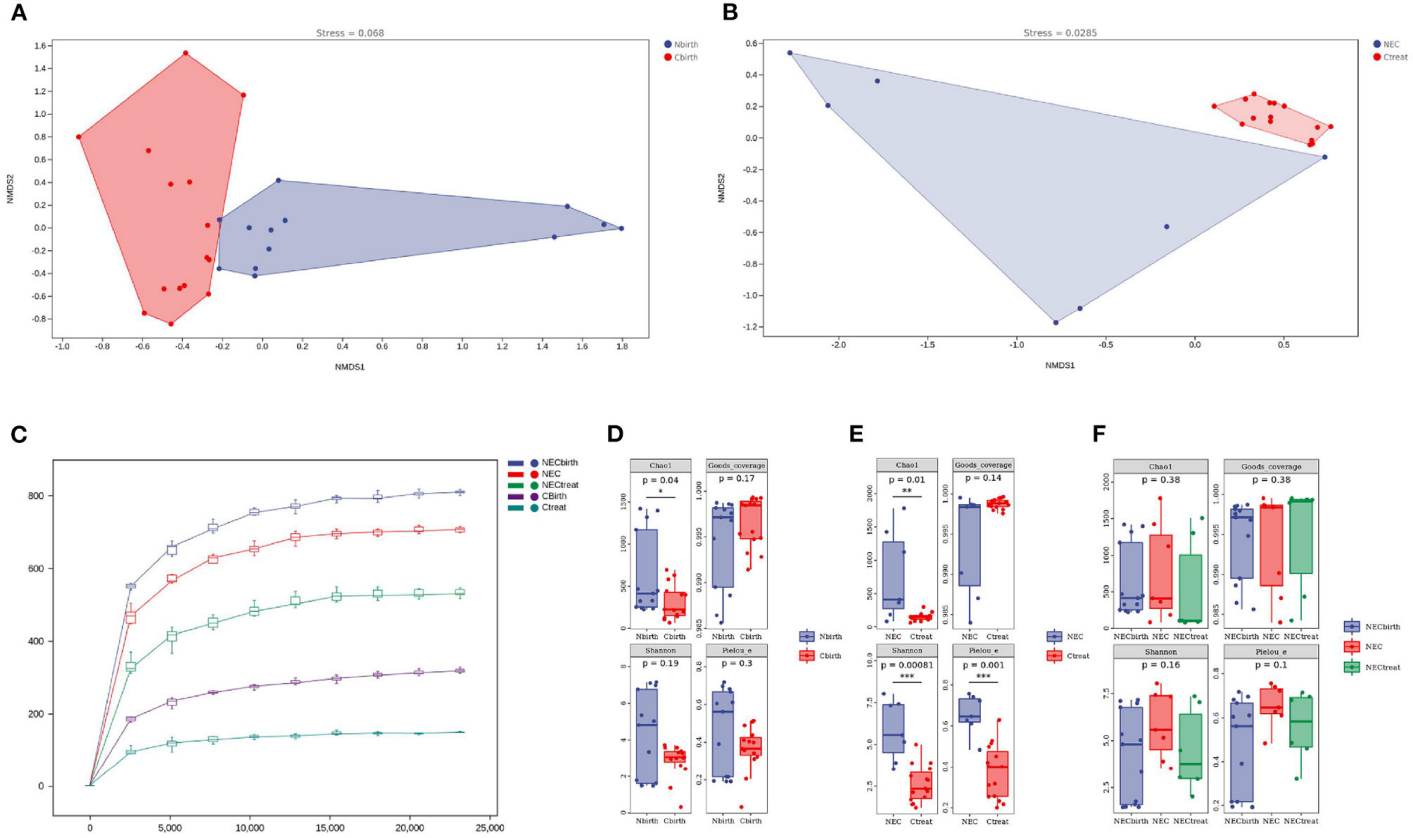
Alpha and beta diversity analysis. **(A,B)** Beta diversity analysis: **(A)** at birth, **(B)** at necrotizing enterocolitis diagnosis. **(C)** Rarefaction curve. **(D–F)** Alpha diversity analysis. Chao 1 index is used to estimate the actual number of species in the community. Shannon diversity index comprehensively considered the richness and evenness of the community. Pielou's_e assesses the coverage of species in the community.

The main purpose of the beta diversity analysis was to investigate the similarity of community structure among different samples. The NMDS of unweighted UniFrac distance was used. The closer (and farther) the distance between two points, the smaller (and greater) the difference in microbial communities between two samples. The Nbirth7 and Nbirth13 samples at birth had an outlier trend. After these two samples were excluded, the control group and the NEC group showed different aggregations at birth (Figure 2A), but the distribution of each sample was relatively scattered. The ANOSIM results suggest values of *R* = 0.264285 and *P* < 0.003. At the onset of NEC, there was obvious aggregation in the control group, while the NEC group was more dispersed and presented subgroups (Figure 2B). Combining the basic information of the specimens showed that the two discrete NEC groups were related to the time of the diagnosis of NEC: in one group, the NEC diagnosis time was more than 20 days, and in the other group, it was <20 days. Moreover, the ANOSIM results indicated values of *R* = 0.812245 and *P* < 0.001 upon NEC occurrence, indicating that the group classification was better. The stress values of the two NMDS results were 0.068 and 0.0285. It is generally believed that, when the value is <0.2, the NMDS analysis result is reliable.

The rarefaction curve tended to be flat after the sequencing depth reached 5,000, indicating that the sequencing results were sufficient to reflect the diversity in the sample (Figure 2C). The diversity index analysis showed that the Chao1 index of the NEC group at birth (Figure 2D) was higher than that of the control group (*P* = 0.04), but the Shannon index (*P* = 0.19), Good's coverage (*P* = 0.17), and Pielou's evenness index (*P* = 0.3) showed no significant difference, indicating that the number of microbes actually present in the NEC group was higher, but the uniformity was not different. Upon the occurrence of NEC (Figure 2E), the Chao1 index (*P* = 0.01), Shannon index (*P* = 0.00081), and Pielou's evenness index (*P* = 0.001) of the NEC group were higher, suggesting that, when NEC occurred in the infants, not only the intestinal microbial richness but also the evenness was higher than that in the control group. This study also performed a dynamic assessment of the stool of NEC infants (Figure 2F) and found that the microbial diversity of the NEC group did not change significantly over time.

**Figure 3:**
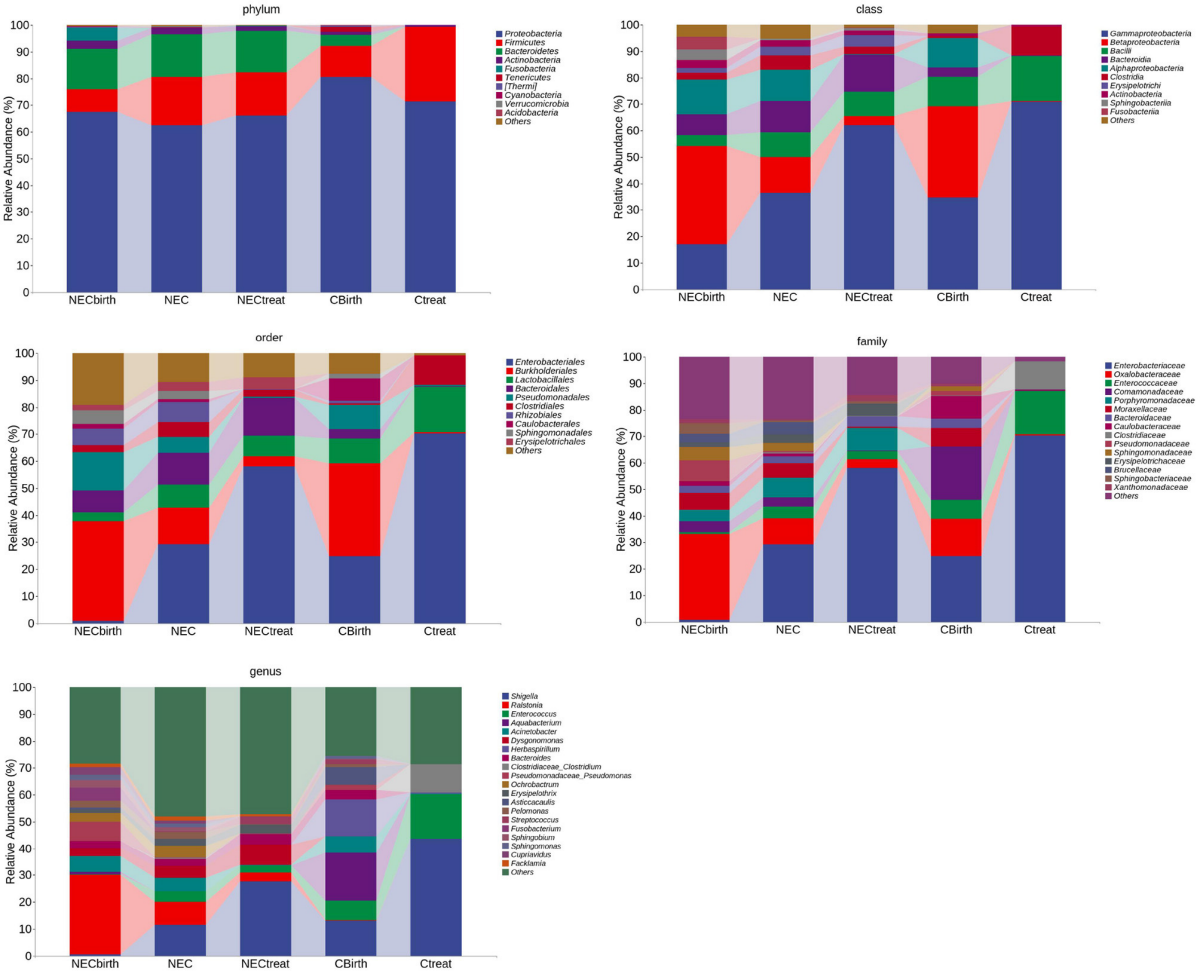
Taxonomic composition analysis. This figure shows the bacteria at the top of each taxonomic level, the top 10 at the phylum, class, and order levels, the top 15 at the family level, and the top 20 at the genus level.

Regardless of whether infants were from the NEC group or control group, the number of dominant species with high abundance in the intestinal flora decreased over time, and a stable flora dominated by fewer species was obtained. The histogram shows that the abundance of Enterobacteriaceae increased over time. The characteristics of the NEC group after treatment were similar to those of the control group (Figure 3).

### Different Flora and Function Prediction

Through LEfSe analysis (logarithmic discriminant analysis threshold of 3), the difference between the NEC and control groups at each taxonomic level was obtained (Supplementary Table 1). While most microbes had low relative abundance, this study analyzed various taxa with high abundance (Figure 3). The results showed that the abundance of Bacteroides and Actinobacteria in NEC infants at birth was much higher than that in the control group, and this trend continued until NEC occurred, with Rhizobiales at the family level and Dysgonomonas, Ochrobactrum, Ralstonia, Pelomonas, Acinetobacter, etc., at the genus level exhibiting the same characteristics. In addition, the NEC group had a higher abundance of Alphaproteobacteria, Betaproteobacteria, Sphingomonas, Lactobacillus, etc., when NEC occurred, while the control group had a significantly higher abundance of Gammaproteobacteria, Enterobacteriaceae, and Clostridiaceae.

**Figure 4:**
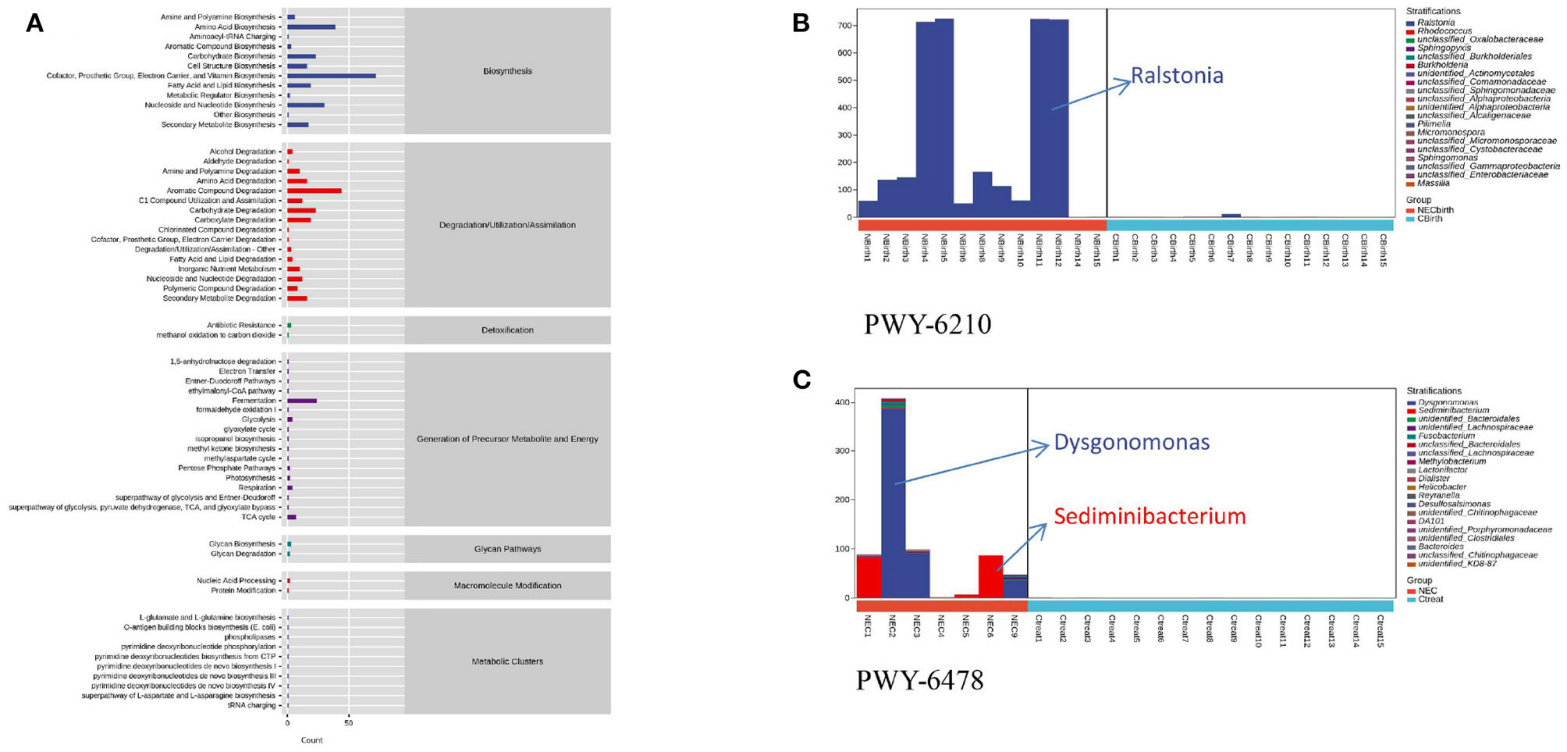
Microbial function prediction. **(A)** Metabolic pathways involving microbes in all infant feces. **(B)** PWY-6210 pathway's species composition. **(C)** PWY-6478 pathway's composition of two species. NEC2, NEC3, and NEC9 are infants diagnosed with necrotizing enterocolitis within 20 days.

PICRUSt2 analysis revealed the metabolic pathways involving microbes in all infant feces (Figure 4A), and the abscissa represents the number of functional pathways—pathways related to degradation/utilization/assimilation; biosynthesis plays a major role. Through comparison, the PWY-6210 and PWY-6505 pathways with statistically significant differences in the NEC group at birth were upregulated and involved in the degradation of L-tryptophan and aromatic compounds, and their species composition was mainly Ralstonia (Figure 4B). The two pathways also remained upregulated within 48 h of NEC diagnosis. At the time of NEC diagnosis, there were 50 upregulated different metabolic pathways in the NEC group (Supplementary Table 2). These pathways were mainly concentrated on degradation/utilization/assimilation, biosynthesis, and generation of precursor metabolites and energy, including the synthesis of sugar, enzyme cofactors, cell structure, fermentation of acetic acid and butyric acid, degradation of lysine and glutamic acid, etc. The species composition included Ralstonia, Dysgonomonas, Bacteroides, and Corynebacterium. At the same time, it was also found that the PWY-6478 pathway was mainly composed of two species, Dysgonomonas and Sediminibacterium, and these two species existed in the feces of infants with different NEC diagnosis times (Figure 4C).

## Discussion

NEC is a serious disease threatening the lives of newborns, but its cause is not truly understood. It is known that intestinal dysbacteriosis, which occurs before the disease appears, is one of the important factors leading to NEC (5). As a large organ formed by microbial aggregation, changes in the intestinal flora need to be further studied in combination with species and community.

### The Overall Perspective of the Bacterial Community

A retrospective study from 2008 to 2015 found that the microbial diversity of most preterm infants with NEC was lower than that of other infants at the same gestational age (6). Moreover, some studies indicated that the Chao1 and Shannon indices were not significantly different between the NEC and control groups, which means that the lack of bacterial diversity was not related to the development of NEC (7–9). This study found that the NEC group had an advantage in the number of microbes at birth. In addition, the richness and evenness of microbes in the NEC group were higher than those in the control group when the disease occurred, which was different from the abovementioned research results. The high microbial diversity of infants with NEC may be related to the greater differences between individuals in the NEC group, while the microbial communities in the control group were more similar. However, regardless of the level of diversity, it only provides different degrees of protection to pathogens and cannot directly induce NEC.

The beta diversity analysis suggested that a difference in the time of NEC diagnosis (with 20 days as the boundary) can also cause differences in the microbial communities of premature infants. Compared with the community at birth, the differences in the communities between the two groups upon the occurrence of NEC were more obvious and more representative. However, in this study, the number of sample cases when NEC occurs is small, and a larger-sample-size study is needed to prove this. A study that analyzed the intestinal flora of infants with early-onset and late-onset NEC (with 22 days as the boundary) indicated that the patterns of microbial progression and dominant colonizing microbes differed between the groups depending on when the infants were diagnosed with NEC (10). PICRUSt2 analysis also shows that early-onset NEC and late-onset NEC are controlled by different species in the PWY-6478 pathway, which further illustrates that the time of NEC occurrence may be related to the different compositions of intestinal microbes. Of course, this difference may also be related to the inconsistent rate of microbial progression caused by the age when the infants' feces were collected. More research is needed to clarify the characteristics of the microflora of infants at different growth stages. A 2014 study found that a variety of factors affect the intestinal flora, but they only affect the rate of progression, not the sequence of development (11). We need to understand how individual microbes change at different stages of the disease to prevent infants from progressing toward the disease through probiotics or other means in the early stage of the disease.

### The Individual Microbes of the Bacterial Community

From a microscopic perspective, recent studies have shown that the main intestinal taxa in premature infants are Proteobacteria, Firmicutes, Bacteroidetes, and Actinobacteria, which play a key role in maintaining intestinal barrier function, increasing the expression of tight junctions, regulating mucin, and affecting biosynthesis. Moreover, the metabolites of these microbes provide energy for the proliferation of epithelial cells and stimulate the immune system (12, 13). The results suggest that the abundances of Bacteroidetes and Acinetobacter are significantly different between the time of birth and NEC onset. Although Bacteroidetes does not have the highest abundance, these species can change their surface structure to escape the host's immune response and produce lysozyme to mediate tissue destruction (14). In addition, in Bacteroides species, the nanH gene can produce neuraminidase to lyse mucin polysaccharides and enhance bacterial growth by producing available glucose. It can also influence the transfer and colonization of other bacterial communities through interleukin-36 (15). Bacteria influence the occurrence and development of disease even when they do not have a high relative abundance. Similarly, Acinetobacter species from Proteobacteria can adhere to human epithelial cells and produce enzymes that destroy tissue lipids. Identifying the characteristics of these microbes could help us to avoid the occurrence of diseases in a targeted manner or reduce the severity of diseases through treatment. In addition, studies have found that mixed infections caused by combinations of other bacteria with Acinetobacter are more toxic than only Acinetobacter infections (16). This suggests that a single strain with superior abundance is not the cause of NEC, and it might be the combined effect of various bacteria that causes NEC. We need to further study which species have higher toxicity and can cause disease and try to avoid the occurrence of combined effects. Acinetobacter is also linked to maternal digestive tract disease during pregnancy, which also reflects that infants' intestinal flora at birth is affected by their mothers (17). By controlling the mothers' disease development during pregnancy, the incidence of neonatal diseases could be reduced.

In addition to the mentioned microbes that mainly cause NEC *via* virulence, some environmental bacteria are more likely to have abundance advantages in the intestines of infants with NEC. For example, Dysgonomonas exists on environmental surfaces in medical institutions (18). Rhizobiales, Ralstonia, and Pelomonas can be detected on the surfaces of pipelines, such as those in hospital water sources and in-hospital ventilators, especially Ralstonia, which can survive even in disinfectants under low nutritional conditions (19–21). These environmentally derived bacteria exhibited colonization within 48 h after the birth of infants with NEC, indicating that premature infants are soon affected by the surrounding environment after leaving their mothers, such as in the operating room, in the neonatal intensive care unit (NICU) or *via* contact with the ventilator pipeline. When NEC occurs, the above-mentioned different flora still exists. Does this mean that the guts of infants with NEC are more likely to be colonized by waterborne bacteria? Targeting these waterborne bacteria or minimizing their exposure to premature babies could reduce the incidence of the disease.

A trend analysis of microbes indicated that the developmental trend in the NEC group after treatment was similar to that in the control group. The abundance of Alphaproteobacteria and Betaproteobacteria showed a downward trend, and Gammaproteobacteria showed an upward trend. It is reasonable to think that, when NEC occurs, the Alphaproteobacteria and Betaproteobacteria in the NEC group showed abundance advantages, which was consistent with the two bacteria being dominant in a large number of water sources and in biofilms, while the content of Gammaproteobacteria is relatively low (21, 22). On the other hand, this phenomenon is also associated with the various factors mentioned above that affect the intestinal flora but only influence the speed of progression, not the sequence of progression.

Functional prediction analysis also suggests that Ralstonia and Dysgonomonas, as the main species of many different metabolic pathways in NEC infants, are mainly involved in the degradation of various amino acids and aromatic compounds. Studies have shown that L-tryptophan participates in intestinal immune homeostasis and can play an anti-inflammatory effect in mammals (23). The L-tryptophan degradation pathway in NEC infants is upregulated both at birth and at the time of disease diagnosis, which reduces the anti-inflammatory effect of premature infants and makes them more prone to disease. Tryptophan is an essential amino acid that can only be ingested through food, and targeted supplementation with tryptophan may reduce the effect of microorganisms on immune homeostasis. In addition, some studies have found that aromatic amino acids can be used as substrates of metabolic pathways to affect intestinal permeability and systemic immunity (24). In-depth exploration of how the flora participates in various metabolic pathways in the human body can help us understand the occurrence of NEC.

There is also an interesting phenomenon that occurred here. Compared with other studies, some of our results are similar, such as Sphingomonas (25), and in some respects opposite, such as Gammaproteobacteria (18). Moreover, the specific microbe and species change trends found in infants with NEC vary from study to study (26–29). This difference may be due to a mixture of factors that alter the microbial community of newborns, such as gestational age, antibiotic treatment of infants and mothers, delivery mode, feeding pattern, and the use of probiotics (30). Additionally, different NICUs and different races will cause different results (31). No study has been able to completely eliminate the effects of these factors. Analysis of the selection criteria for the participants in each study showed that most of the studies excluded infants who received probiotics, and the selection of gestational age for infants varied from study to study. The different gene target regions (V1–V3 or V3–V5), different analysis platforms analyzed in 16sRNA sequencing technology, different NICU treatment plans, and environmental and ethnic differences—all of these factors—could have influenced the results of the study. Microorganisms carried in breast milk cannot be detected, which is an uncharted territory.

### Limitations

Due to the particularity of the disease, only 15 premature infants with NEC were included in this study. The antibiotic utilization rate in premature infants was high, and it was difficult to acquire a sample before antibiotics were applied, resulting in influencing factors that could not be ruled out. This effect can only be reduced by matching the antibiotic application time of the two groups of children. Our study failed to use metagenomic sequencing technology to analyze the link between the intestinal flora and NEC, and more in-depth research is needed. In the comparison of the microbiomes of NEC patients, the inconsistency in age of onset of the disease is a confounding factor, and the relationship among antibiotics, delivery type (vaginal or cesarean section), diet, and infant age affects the rate of microbiota progression. Moreover, infants need to fast for a short duration after illness, which increases the difficulty of sample retention. This study was limited to a single research center, and more studies are needed to verify the conclusions.

### Conclusions

1. Based on the time of NEC diagnosis (bounded by 20 days), the microbial community differs.
2. No single microorganism is related to NEC, and the combined effect of multiple species is of great significance in the occurrence of NEC. Premature infants are easily affected by bacteria living in the environment, and compared with ordinary premature infants, NEC infants have a higher abundance of waterborne bacteria, so attention should be paid to the contamination of water sources and various ventilator pipelines for premature infants hospitalized in the NICU.
3. An in-depth study of the mode of microbial colonization in premature infants, combined with the different functions of various metabolic pathways involved in different microorganisms, may be able to identify the cause of NEC.

## Data Availability Statement

The datasets presented in this study can be found in online repositories. The names of the repository/repositories and accession number(s) can be found in the article/Supplementary Material.

## Ethics Statement

The studies involving human participants were reviewed and approved by the Ethics Committee of the First Hospital of Jilin University (Ethics No. 2018-421). Written informed consent to participate in this study was provided by the participants' legal guardian/next of kin.

## Author Contributions

XF and SL contributed to the study design, data collection, data analysis, and manuscript writing. YJ an XH made contributions to the methodology and data interpretation. HW revised the manuscript and approved the final version. All the authors approved the final manuscript as submitted and agree to be accountable for all aspects of the work.

## Conflict of Interest

The authors declare that the research was conducted in the absence of any commercial or financial relationships that could be construed as a potential conflict of interest.
